# Effectively Converting Cane Molasses into 2,3-Butanediol Using *Clostridium* *ljungdahlii* by an Integrated Fermentation and Membrane Separation Process

**DOI:** 10.3390/molecules27030954

**Published:** 2022-01-30

**Authors:** Yuling Yang, Tingting Deng, Weifeng Cao, Fei Shen, Sijia Liu, Jing Zhang, Xinquan Liang, Yinhua Wan

**Affiliations:** 1 Department of Sugar Engineering, College of Light Industry and Food Engineering, Guangxi University, Nanning 530004, China; yangyulingmie@163.com (Y.Y.); lsj53158352@163.com (S.L.); zhangjing0542@163.com (J.Z.); 2 State Key Laboratory of Biochemical Engineering, Institute of Process Engineering, Chinese Academy of Sciences, Beijing 100190, China; fshen@ipe.ac.cn (F.S.); yhwan@ipe.ac.cn (Y.W.); 3 Agro-Product Safety Research Center, Chinese Academy of Inspection and Quarantine, Beijing 100123, China; angellove0405@163.com; 4 School of Chemical Engineering, University of Chinese Academy of Sciences, Beijing 100049, China

**Keywords:** 2,3-butanediol, *Clostridium ljungdahlii*, cane molasses, membrane separation

## Abstract

Firstly, 2,3-butanediol (2,3-BDO) is a chemical platform used in several applications. However, the pathogenic nature of its producers and the expensive feedstocks used limit its scale production. In this study, cane molasses was used for 2,3-BDO production by a nonpathogenic *Clostridium ljungdahlii*. It was found that cane molasses alone, without the addition of other ingredients, was favorable for use as the culture medium for 2,3-BDO production. Compared with the control (i.e., the modified DSMZ 879 medium), the differential genes are mainly involved in the pathways of carbohydrate metabolism, membrane transport, and amino acid metabolism in the case of the cane molasses alone. However, when cane molasses alone was used, cell growth was significantly inhibited by KCl in cane molasses. Similarly, a high concentration of sugars (i.e., above 35 g/L) can inhibit cell growth and 2,3-BDO production. More seriously, 2,3-BDO production was inhibited by itself. As a result, cane molasses alone with an initial 35 g/L total sugars was suitable for 2,3-BDO production in batch culture. Finally, an integrated fermentation and membrane separation process was developed to maintain high 2,3-BDO productivity of 0.46 g·L^−1^·h^−1^. Meanwhile, the varied fouling mechanism indicated that the fermentation properties changed significantly, especially for the cell properties. Therefore, the integrated fermentation and membrane separation process was favorable for 2,3-BDO production by *C. ljungdahlii* using cane molasses.

## 1. Introduction

Firstly, 2,3-butanediol (2,3-BDO) is a valuable chemical feedstock due to its potential applications, including its use as a liquid fuel and for the production of various chemicals, foods, and pharmaceuticals [1,2,3,4]. The global market of the BDO is expected to reach a value of USD 220 million by 2027, growing at a compound annual growth rate (CAGR) of 3% from 2019 to 2027 [5], and the current market price of 2,3-BDO is higher than that of 1,4-butanediol [6]. The production of 2,3-BDO through a bioprocess is an attractive aim, given its environmental friendliness. In addition, 2,3-BDO can be produced by many wild-type microorganisms, such as *Klebsiella pneumoniae* [7], *Klebsiella oxytoca* [8], and *Serratia marcescens* [9]. However, the pathogenic nature of *K. pneumoniae* and *S. marcescens*, which are defined as risk class 2 organisms, limits their utilization for industrial-scale production processes [10]. Thus, one approach of choice is to screen new class 1 microorganisms on selective media dedicated to safe microflora.

The fermentation of excess biomass or waste from agricultural and agro-industrial residues to produce 2,3-BDO has gained considerable attention due to the forthcoming scarcity of traditional fuels, as well as the need for more reasonable management of food and feed sources. To date, much effort has been made to find inexpensive feedstocks, such as corncob [11,12], jatropha hulls [13], jerusalem artichoke tubers [14], molasses [15,16,17], corn stover hydrolysate [18], sugarcane bagasse hydrolysates [3], and oil palm empty fruit bunches [7]. Among the feedstocks, sugarcane molasses is an important industrial carbon source composed of sucrose, glucose, fructose, nitrogenous substances, vitamins, and trace elements [19], and is considered to be a promising feedstock for biorefineries due to its rich sugar content and cost-effectiveness. In addition, the quantity of cane molasses available in China is approximately 4,800,000 tons/year, and the price of cane molasses is lower than USD 150/ton [20]. By supplementing glycerol-containing medium with 10 g/L of sugarcane molasses, the final 2,3-BDO production was enhanced to 19 g/L by *Paenibacillus polymyxa*, a GRAS (generally recognized as safe) bacterium [21]. However, when provided with a mixture of different carbon sources, most microorganisms prefer to use one carbon source for the fastest growth, and the presence of preferred carbon sources often prevents the utilization of secondary substrates [16]. Thus, it is urgent to study whether a GRAS bacterium can effectively assimilate molasses for 2,3-BDO production.

For 2,3-BDO production, the hexoses enter the microbial metabolism through PTS (phosphortransferase system) and convert into 2 moles of pyruvate via the Embden–Meyerhof pathway (glycolysis) with the generation of 2 moles of NADH and 2 moles of ATP. Pyruvate is then channeled for the generation of metabolites such as acetate, formate, lactate, succinate, ethanol, and acetoin, besides BDO [5]. Thus far, little is known about the functional aspect of the metabolic regulation of 2,3-BDO production. It is, however, hypothesized that 2,3-BDO biosynthesis has essential physiological importance, including preventive acidification, contributing to cell regulation of the NADH/NAD^+^ ratio and the storage of carbon and energy for microbial growth [1]. Nowadays, nonpathogenic microorganisms belonging to risk group 1 are the most desirable biocatalysts, such as *P. polymyxa*, *Raoultella* strains, and *Bacillus* strains. In shaken flasks, approximately 14.5, 16.8, and 8.0 g/L 2,3-BDO was produced by *P. polymyxa* [21], *Bacillus amyloliquefaciens* B10-127 [22], and *Raoultella terrigena* CECT 4519 [23], respectively. Among the strains, 2,3-BDO metabolism is dependent on strict oxygen control to ensure an oxygen supply condition, and oxygen supply is considered one of the most critical factors in 2,3-BDO production, affecting the distribution of metabolites produced, yield, and productivity [5,10]. However, oxygen supply is also a high-cost process that requires the supply of compressed air or pure oxygen. Recently, it was reported that 2,3-BDO is one product in the strictly anaerobic fermentation of *Clostridium ljungdahlii* [24], where the supply of oxygen must be eliminated in the broth. *C. ljungdahlii* is a common soil bacterium found all over the world, and it is also an important industrial biocatalyst. The first strain ever isolated (PETC ATCC 49587/ ATCC 55383) was from chicken yard waste and the second (ERI2 ATCC 55380) from a natural water source [25]. This Gram-positive, motile, spore-forming rod’s metabolism is primarily acetogenic. *C. ljungdahlii* grows with carbon monoxide, hydrogen and carbon dioxide, ethanol, pyruvate, arabinose, xylose, fructose, or glucose. *C. ljungdahlii* is the first acetogen in clostridial 23S rRNA homology group I [26], and is a promising platform organism for syngas fermentation [24]. In addition, *C. ljungdahlii* is defined as a risk class 1 organism. Thus, the use of *C. ljungdahlii* as a producer for 2,3-BDO production may be more favorable. Moreover, the use of molasses as a feedstock for 2,3-BDO production using *C. ljungdahlii* needs to be evaluated.

In the study reported here, the aim was to evaluate the production of 2,3-BDO via microbial fermentation by *C. ljungdahlii* from cane molasses. Furthermore, an integrated fermentation and membrane separation process was used to enhance 2,3-BDO productivity.

## 2. Results and Discussion

### 2.1. Assimilation of Sugars in the Molasses by C. ljungdahlii DSM 13528 for 2,3-BDO Production

**Figure 1:**
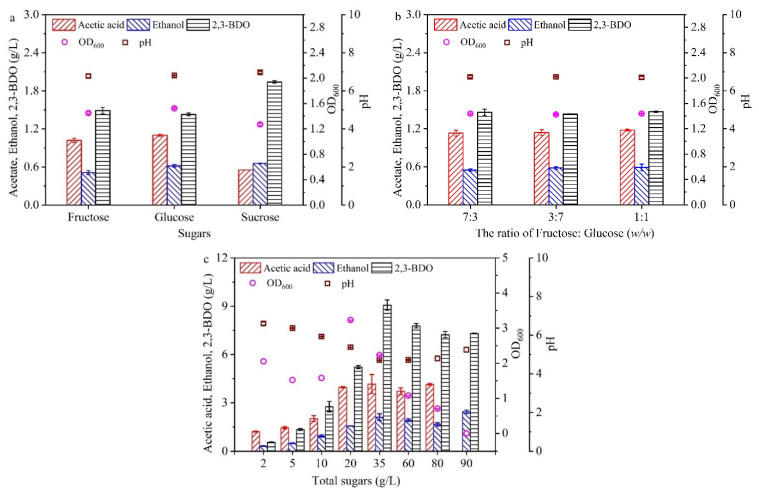
Effect of sugars (**a**), the ratio of fructose:glucose (**b**), and total sugars in molasses (**c**) on 2,3-BDO production. The basal medium was the same as the modified DSMZ 879 medium, while the sugar (i.e., fructose) was substituted by a different substrate in Figure 1. The fermentation was carried out in 250 mL screw-cap bottles. Data are given as the mean ± SD, n = 3.

To clarify the mechanism of sugar assimilation by *C. ljungdahlii* DSM 13528 for 2,3-BDO production using molasses, a series of experiments using fructose, glucose, and sucrose as substrates were first performed (Figure 1a), where the sugar concentration (i.e., 5 g/L) was the same as that in the modified DSMZ 879 medium. As expected, fructose, glucose, and sucrose could all be assimilated by the strain DSM 13528 to produce 2,3-BDO, and acetic acid and ethanol were the main by-products. The 2,3-BDO concentrations were 1.94, 1.49, and 1.43 g/L from sucrose, fructose, and glucose, respectively, which indicated that sucrose was the more suitable substrate for 2,3-BDO production, and fructose and glucose gave similar results for 2,3-BDO production. The cell growth (OD_600_) was 1.27, 1.45, and 1.52 from sucrose, fructose, and glucose, respectively. This indicated that fructose and glucose gave similar results for cell growth and were more suitable substrates for cell growth. The ethanol concentrations were 0.656, 0.511, and 0.619 g/L from sucrose, fructose, and glucose, respectively. The acetic acid levels were 0.533, 1.02, and 1.1 g/L from sucrose, fructose, and glucose, respectively, which indicated that glucose was the more suitable substrate for acetic acid production. These results indicated that the strain DSM 13528 utilized the sugars differently for 2,3-BDO production and cell growth. Moreover, the molasses simultaneously contained sucrose, fructose, and glucose, where the sucrose was the main ingredient [27,28]. However, for 2,3-BDO production, the sugar mixture in molasses is not consumed as efficiently as glucose by *E. aerogenes* due to complex interactions among their utilizing pathways, such as carbon catabolite repression (CCR) [16]. In addition, most of the sucrose was not taken up through a direct transport system, but was converted to glucose and fructose [29]. Thus, the effect of the mixed sugars with different fructose:glucose ratios on 2,3-BDO production needs to be explored.

To evaluate the effect of the fructose:glucose ratio on 2,3-BDO production, a mimic sugar mixture was first used. As shown in Figure 1b, the fructose:glucose ratio had little effect on 2,3-BDO production, cell growth, and by-product production (<5%). It indicated that the strain DSM 13528 has a high capacity for sugar assimilation, differing from *E. aerogenes* [16]. Then, different concentrations of the total sugars (i.e., the sum of sucrose, glucose, and fructose) in real molasses were used to replace the sugar (i.e., fructose) in the modified DSMZ 879 medium (Figure 1c). When the concentration of total sugars was 5 g/L, the concentrations of 2,3-BDO, ethanol, and acetic acid were 1.34, 0.50, and 1.45 g/L, respectively, where the acetic acid concentration was highest in the products. The ratio of the products in the broth was very different from that using the single sugar (Figure 1a) or mimic sugar mixture (Figure 1b), which may have been affected by other ingredients in the molasses. Further decreasing the concentration of total sugars (i.e., 2 g/L), the ratio of acetic acid was further enhanced. In addition, the synthesis of 2,3-BDO was regarded as a carbon- and energy-storing strategy, and 2,3-BDO can be reutilized during the stationary phase when other carbon and energy sources have been depleted [30]. However, further increasing the concentration of total sugars above 5 g/L, the ratio of 2,3-BDO in the broth was significantly increased, and the maximal 2,3-BDO concentration of 9.1 g/L was obtained at 35 g/L total sugars. This indicated that the concentration of total sugars in molasses affected not only the 2,3-BDO production but also the ratio of 2,3-BDO in the products. 

Moreover, in addition to a large amount of sugars, molasses contain nitrogenous substances, vitamins, and trace elements [19]. Meanwhile, the modified DSMZ 879 medium also contains nitrogenous substances, vitamins, and trace elements [24]. Thus, it is necessary to evaluate whether some ingredients in the modified DSMZ 879 medium could be replaced by those in molasses.

### 2.2. Replacement of Ingredients in the Modified DSMZ 879 Medium Using Molasses 

**Figure 2:**
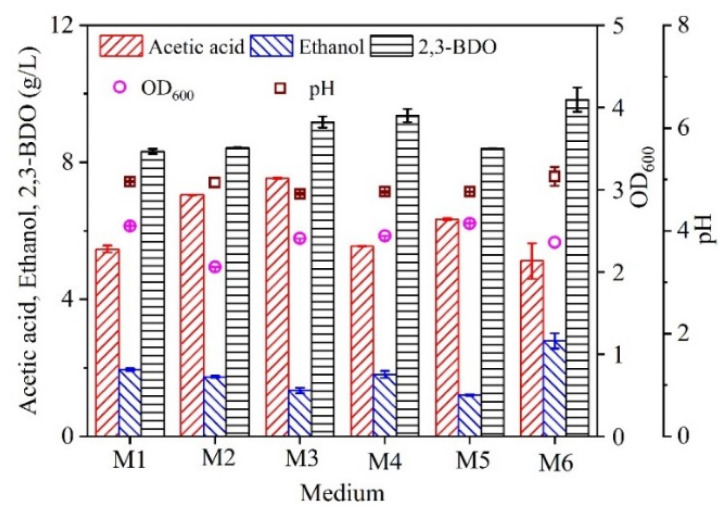
Effect of fermentation medium on 2,3-butanediol production. The components of M1, M2, M3, M4, M5, and M6 were shown in Table 1. The fermentations were carried out in 250 mL screw-cap bottles. Data are given as the mean ± SD, n = 3.

In order to evaluate the feasibility of using molasses to replace the ingredients in the modified DSMZ 879 medium for 2,3-BDO production, different parts of the ingredients in the modified DSMZ 879 medium were added to the molasses containing 35 g/L total sugars (i.e., medium M6 in Table 1), since the highest concentration of 2,3-BDO was obtained at the value of 35 g/L total sugars from the real molasses (Figure 1c). As shown in Figure 2, when Part A, Part B, and Part C in the modified DSMZ 879 medium were added to medium M6 (i.e., medium M4 in Table 1), the concentration of ethanol was decreased by 35.1% compared with that in M6, the concentration of acetic acid was increased by 8.4%, while the concentration of 2,3-BDO showed no major differences by statistical analysis (< 5%). This indicated that all the ingredients in the modified DSMZ 879 medium could be replaced with molasses for 2,3-BDO production. Furthermore, when Part A, Part B, or Part C was added to the medium M6, the concentrations of 2,3-BDO and ethanol were decreased compared with those in medium M6, and the concentration of acetic acid was increased. This suggested that no ingredients in the modified DSMZ 879 medium needed to be added to medium M6 for 2,3-BDO production. However, compared with that in medium M4 or M6, the concentration of acetic acid was highest in medium M3, which meant that the trace element solution (i.e., Part B) in the modified DSMZ 879 medium could induce the carbon metabolism to shift for acetic acid production. In addition, when Part A in the modified DSMZ 879 medium was added to M6 (i.e., medium M1 in Table 1), the concentrations of 2,3-BDO and ethanol were decreased by 15.4% and 30.1%, respectively, compared with those in M6, while the concentration of acetic acid was increased by 6.8%. Part A contained a variety of inorganic salt ions. Similarly, medium M6 (i.e., the molasses containing 35 g/L total sugars) also contained a lot of salt ions [28], such as 2.4 g/L K^+^, 1.1 g/L Cl^−^, 1.32 g/L SO_4_^2−^, 0.23 g/L Na^+^, 0.19 g/L Ca^2+^, and 0.74 g/L Mg^2+^. If a fed-batch culture was carried out, the salt ions from the molasses would be accumulated in the broth, which may decrease the 2,3-BDO production. Thus, in order to produce a high concentration of 2,3-BDO, it is necessary to further evaluate the effect of molasses on 2,3-BDO production with batch and fed-batch cultures.

### 2.3. Effect of Batch and Fed-Batch Cultures on 2,3-BDO Production with the Sole Molasses as the Substrate

**Figure 3:**
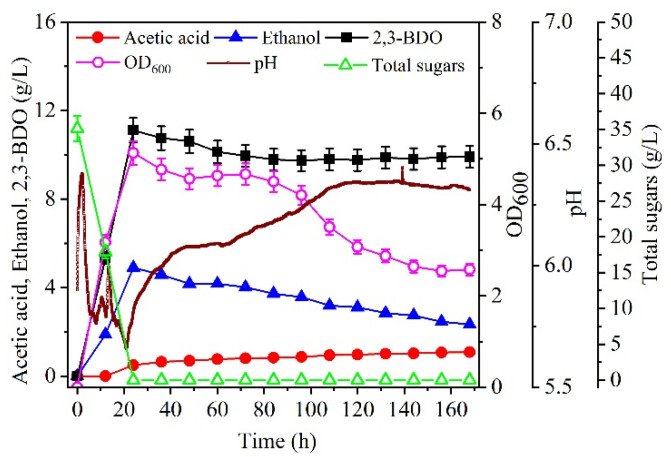
Effect of batch culture on 2,3-BDO production. The cultures were carried out in a 2.7 L bioreaction. Data are given as the mean ± SD, n = 2.

**Figure 4:**
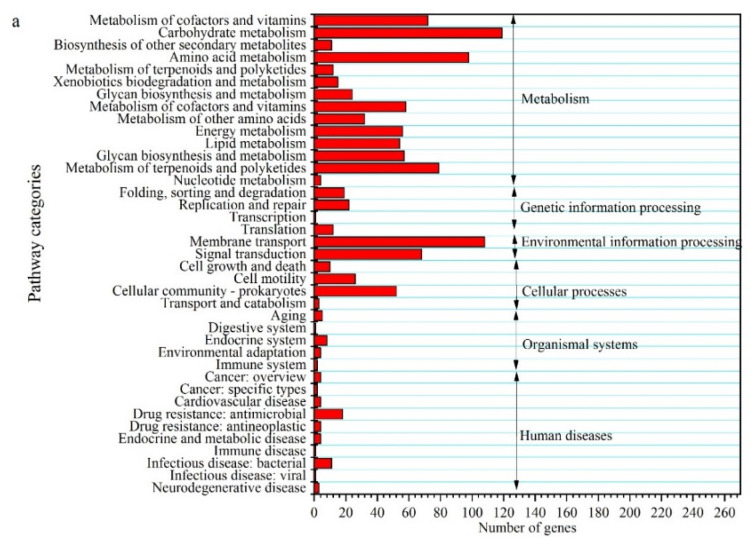

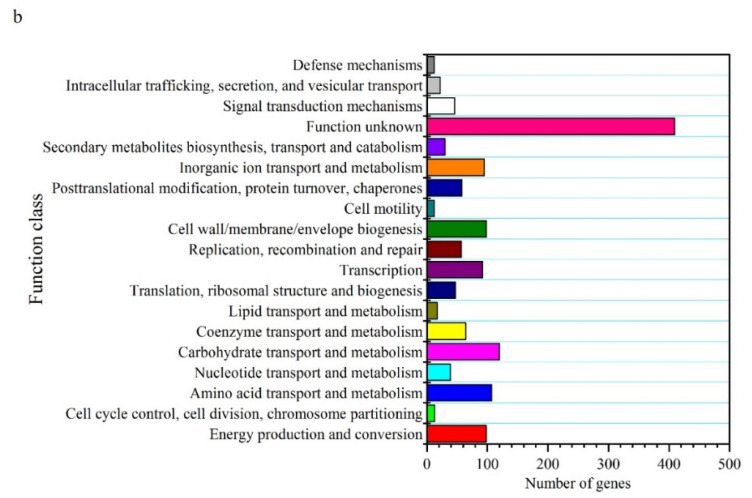
KEGG functional classification (**a**) and COG functional classification (**b**) of the differential genes. The differential genes between fructose were used as the carbon source in the modified DSMZ 879 medium (control) and cane molasses alone was used as the fermentation medium.

When an initial total sugar concentration of 35 g/L was used in a batch culture (Figure 3), the maximal concentrations of 2,3-BDO and ethanol were 11.1 and 4.90 g/L, respectively. They were obtained at 24 h, when the total sugars were depleted. In addition, to further evaluate whether the metabolic pathways shifted, the differential genes between fructose were used as the carbon source in the modified DSMZ 879 medium (control) and cane molasses alone was used as the fermentation medium (Figure 4). According to KEGG and COG analysis, it was found that the differential genes are mainly involved in the pathways of carbohydrate metabolism, membrane transport, and amino acid metabolism. After 24 h, the concentrations of 2,3-BDO and ethanol decreased, and the cell concentration also decreased rapidly. However, the concentration of acetic acid increased until the end of fermentation, at the expense of 2,3-BDO and ethanol. This indicated that the strain DSM 13528 could assimilate the 2,3-BDO and ethanol for acetic acid production, which may have resulted in the different ratios of the products shown in Figure 2. Thus, we assumed that more 2,3-BDO or ethanol could be produced with a fed-batch culture to maintain a high sugar concentration after 24 h, which also prevented 2,3-BDO and ethanol from assimilating by the strain DSM 13528. 

**Figure 5:**
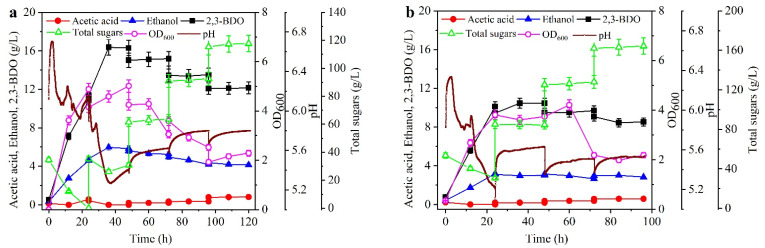
Effect of fed-batch culture on 2,3-BDO production. The time courses refer to (**a**) an initial 35 g/L total sugars with a final concentration of 35 g/L total sugars in the broth from each fed-solution, and (**b**) an initial 55 g/L total sugars with a final concentration of 55 g/L total sugars in the broth from each fed-solution. The cultures were carried out in a 2.7 L bioreaction. Data are given as the mean ± SD, n = 2.

Since the total sugars can be depleted by the strain DSM 13528 in 24 h, a new final concentration of 35 g/L total sugars was fed into the broth every 24 h. As shown in Figure 5a, the maximal concentrations of 2,3-BDO and ethanol were 16.4 and 5.98 g/L, respectively, which were obtained at 36 h. After 36 h, no new 2,3-BDO was produced, where the decreased 2,3-BDO in each fed-batch was caused by the increased volume of the broth. Unexpectedly, only 9 g/L sugars were assimilated in the first feed stage, and no sugars were assimilated in the following feed stages. Meanwhile, the cell concentrations decreased rapidly after 48 h. This indicated that a large amount of 2,3-BDO can only be produced in the initial batch stage. Thus, we further assumed that more 2,3-BDO could be produced in a fed-batch culture with a high initial concentration of sugars (i.e., 55 g/L), and thus a new final concentration of 55 g/L total sugars was fed into the broth every 24 h, which could supply enough substrate in the initial batch stage. As shown in Figure 5b, the concentrations of 2,3-BDO and ethanol were only 10.1 and 3.19 g/L at 24 h, respectively. Compared with those in Figure 5a at 24 h, the concentrations of 2,3-BDO and ethanol in Figure 5b show reductions of 9.9% and 34.9%, respectively, and the sugar consumption rate in Figure 5b decreased by 35.3%. After 24 h, no sugar was consumed, similarly to the result in Figure 5a after 48 h. Since the molasses alone was used as the culture medium, when the concentration of sugars in the molasses varied, the concentration of the cations or anions in the molasses varied according to the sugar concentration. Thus, we cannot confirm that the inhibition effect resulted from substrate inhibition (i.e., sugars) or cations or anions in the molasses. In addition, considering that a lower concentration of 2,3-BDO was produced when the total sugars was above 35 g/L (Figure 1c), there may be some inhibitory factors for 2,3-BDO production in the fed-batch culture or batch culture, such as a high concentration of salts from molasses, high concentration of total sugars, or product inhibition from 2,3-BDO and acetic acid, which will be evaluated in the following section.

### 2.4. Effect of the Inhibitory Factors on 2,3-BDO Production with Molasses Alone as the Substrate

**Figure 6:**
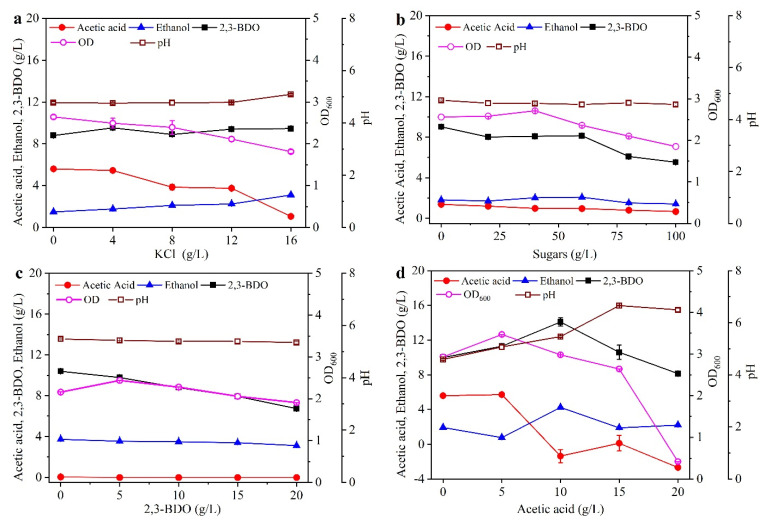
Effect of exogenous KCl (**a**), sugars (**b**), 2,3-BDO (**c**), and acetic acid (**d**) on 2,3-BDO production. The basal medium was the same as that in Figure 1c with 35 g/L sugars in molasses. In (**b**), the sugars in the horizontal axis mean the simulated sugars in molasses, which were further added to the initial 35 g/L real molasses solution in Figure 1c. In (**c**,**d**), the concentration of 2,3-BDO or acetic acid in the vertical axis means the final concentration of 2,3-BDO or acetic acid in the broth subtracted its initial concentration in the medium. The fermentations were carried out in 250 mL screw-cap bottles. Data are given as the mean ± SD, n = 3.

Because K^+^ and Cl^-^ were the main cation and anion in molasses [28], respectively, KCl was used to estimate the effect of salts on 2,3-BDO production. As shown in Figure 6a, the cells and acetic acid concentrations both decreased with the enhanced KCl concentration, and ethanol production showed the reverse trend, while the KCl concentration showed little effect on 2,3-BDO production (<5%). Thus, the salts were not the direct factors for the decrease in 2,3-BDO concentration in the fed-batch culture. Moreover, since a higher initial sugar concentration resulted in lower 2,3-BDO production, as in Figure 1c, the sugar concentration was also evaluated for its effect on 2,3-BDO production. It was found that the 2,3-BDO concentration decreased with the enhanced exogenous sugar concentration, and the 2,3-BDO concentration was decreased by 38.8% after the addition of exogenous 100 g/L compared with the case with no addition. The cell concentration increased slightly when the exogenous sugar concentration was below 40 g/L, while its concentration decreased quickly when the exogenous sugar concentration was above 40 g/L. For example, compared with no addition of exogenous sugars, the cell concentration increased by 5.86% with the addition of 40 g/L exogenous sugars, while its concentration decreased by 27.7% after the addition of 100 g/L exogenous sugars. Thus, a higher concentration of sugars would decrease not only the cell growth but also the 2,3-BDO production, which is similar to the results in Figure 1c and Figure 5. In addition, the inhibition of end-products (i.e., 2,3-BDO and acetic acid) was further evaluated. As shown in Figure 6c, the exogenous 2,3-BDO significantly inhibited its production, but had little effect on the production of acetic acid or ethanol (<5%). For example, compared to no addition of exogenous 2,3-BDO, the concentration of 2,3-BDO was decreased by 35.2% after the addition of 20 g/L exogenous 2,3-BDO. Another product, acetic acid, significantly inhibited its own production. When the exogenous acetic acid concentration was below 10 g/L, it increased 2,3-BDO production, while it inhibited 2,3-BDO production significantly when its concentration was above 10 g/L. For example, compared to no addition of exogenous acetic acid, the 2,3-BDO concentration was increased by 41.0% with the addition of 10 g/L exogenous acetic acid, while its concentration was decreased by 18.6% with the addition of 20 g/L exogenous acetic acid. In fact, it was reported that 2,3-BDO synthesis was induced under acid supplementation [31], which may suggest that 2,3-BDO, as a neutral metabolite, counteracted the excessively high acidification. On the other hand, most studies on the influence of acid supplementation on 2,3-BDO synthesis were performed with acetic acid [32], which is known to induce enzymes involved in the 2,3-BDO pathway [33]. Similarly, when the exogenous acetic acid concentration was below 5 g/L, it increased the cell growth, while it inhibited cell growth significantly when its concentration was above 5 g/L. Considering that the acetic acid concentration was below 1.0 g/L in the broth (Figure 3), the inhibiting effect of acetic acid on 2,3-BDO production was omitted. Similarly, it was reported that, under acidic conditions, dissociated acetic acid cannot easily diffuse across the plasma membrane to the cytosol [34]. Thus, the inhibiting effect of acetic acid could be relieved at a lower acetic acid concentration. Moreover, since the strain DSM 13528 could produce up to 32.8 g/L ethanol [24] and the highest ethanol concentration was below 4.9 g/L, as in Figure 3, the effect of exogenous ethanol on 2,3-BDO production was not taken into account. Therefore, considering the results in Figure 1, Figure 3, and Figure 5, the suitable initial sugar concentration for 2,3-BDO production was 35.0 g/L, and the produced 2,3-BDO would significantly inhibit its own production.

### 2.5. Production of 2,3-BDO Using an Integrated Fermentation and Membrane Separation Process

**Figure 7:**
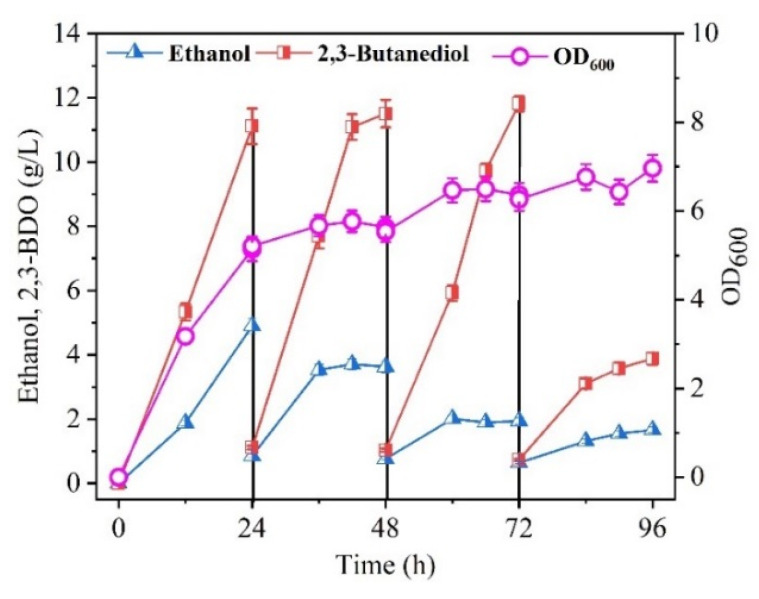
Kinetic curves of 2,3-BDO production in a membrane bioreactor with repeated-batch culture. Data are given as the mean ± SD, n = 2.

Considering the results in Figure 5, the high concentration of 2,3-BDO produced in the broth was a possible reason for the lower production of 2,3-BDO in the fed-batch culture (Figure 5). For bioprocess engineering, it was reported that the productivity of succinic acid [35], L-lactic acid [36], and β-poly(malic acid) [37] could be enhanced in a membrane bioreactor with cell recycling by removing the higher product to order to eliminate its inhibitory effect. Thus, 2,3-BDO productivity may be enhanced by removing the high concentration of 2,3-BDO after 35 g/L molasses depletion. For this purpose, a 300 kDa membrane for recycling cells of *C. ljungdahlii* and removing the produced 2,3-BDO from the broth was performed. As shown in Figure 7, the repeated-batch culture was carried out for three cycles, in which a similar 2,3-BDO titer (11.1 g/L) and productivity (0.46 g/L·h) were achieved in the first two cycles. However, the 2,3-BDO titer decreased significantly in the third cycle. Compared with the batch culture, the 2,3-BDO productivity was not significantly enhanced in this repeated-batch culture. However, the seed culture time (48 h) was eliminated in this process. Moreover, the cell recirculation with the membrane system did show a higher cell yield, while the specific 2,3-BDO production per unit cell mass (Y_p/x_) increased in succession with the recycled time. Thus, two cycles of repeated batches in the integrated fermentation and membrane separation process were more suitable. In the first two cycles and the initial batch culture, the average yield of 2,3-BDO from the total sugars was 0.32 g/g, which was higher by 10.3% than that from another nonpathogenic microorganism, *P. polymyxa* PM 3605, from glycerol [21]. However, the 2,3-BDO production and yield were much lower than those from another pathogenic microorganism, *K. pneumoniae* PM2 [7]. In addition, although *C. acetobutylicum* can produce 1,4-BDO [38], it does not produce 2,3-BDO because of the absence of an acetoin reductase [39]. However, *C. ljungdahlii* provides the acetoin reductase [40]. To the best of our knowledge, this is the first time that 2,3-BDO has been produced by *C. ljungdahlii* from cane molasses. However, the production of 2,3-BDO was still lower than the production with a pathogenic microorganism. According to the results from Figure 4, metabolic engineering of *C. ljungdahlii* to remodel the metabolic pathways of carbohydrate metabolism, membrane transport, and amino acid metabolism may be possible to enhance 2,3-BDO production. In fact, some metabolically engineered strains, such as *Saccharomyces cerevisiae* [41], *Enterobacter aerogenes* [3], and *Paenibacillus polymyxa* [42], have been constructed for producing high concentrations of 2,3-BDO.

**Figure 8:**
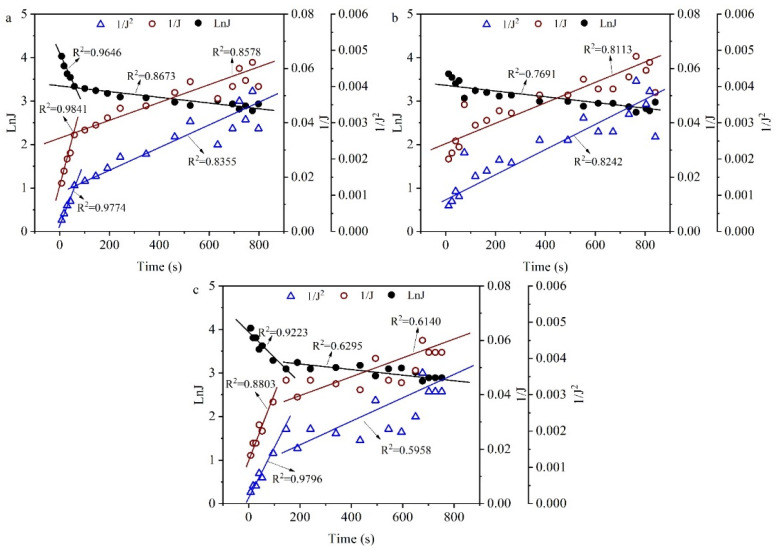
Determination of fouling mechanisms from model fitting to experimental data in Figure 7: (**a**) cycle 1, (**b**) cycle 2, and (**c**) cycle 3.

Furthermore, the membrane fouling mechanism during filtration of the broth was unraveled (Figure 8). Flux decay rates were greatest at the beginning in all cases and then continued to decline slowly, without a clear steady state. This indicated that fouling occurred rapidly once the feed solution contacted the membrane module. As mentioned earlier [43,44], fouling mechanisms could be identified by fitting the experimental data to typical fouling models. The results are presented in Figure 6, and by comparing their regression coefficients, the best possible fouling mechanism for each feed solution can be determined. It is found that the main fouling mechanism in the initial stage during filtration of the broth was the membrane resistance, and then pore blocking became the main source of resistance in cycle 1. In cycle 2, the main fouling mechanism was cake formation. However, in cycle 3, the main fouling mechanism in the initial stage during filtration of the broth was cake formation, and then pore blocking became the main source of resistance. The varied fouling mechanisms indicated that the fermentation properties changed significantly, especially for the cell properties. Recently, it was reported that a reverse osmosis membrane showed the potential to separate 2,3-BDO from the actual fermentation broth [45]. Thus, the obtained broth after filtering with the 300 kDa membrane was favorable for the downstream processing of 2,3-BDO.

## 3. Materials and Methods

### 3.1. Microorganism, Media, and Cultivation Conditions

*C. ljungdahlii* DSM 13528, cultivation cond itions, and the modified DSMZ 879 medium were the same as those reported in Zhu et al. [24] (refer to the Supplementary Information for details of the modified DSMZ 879 medium). The modified DSMZ 879 medium had the following composition (per liter): 1.0 g NH_4_Cl, 0.1 g KCl, 0.2 g MgSO_4_·7 H_2_O, 0.8 g NaCl, 0.02 g CaCl_2_·2 H_2_O, 0.1 g KH_2_PO_4_, 2.5 mg Na_2_WO_4_·2 H_2_O, 1.0 g NaHCO_3_, 1.0 g cysteine-HCl·H_2_O, 1 g yeast extract, 0.5 g cysteine, 0.5 mg resazurin (Part A); 10 mL trace element solution (Part B); 10 mL vitamin solution (Part C); and 5.0 g fructose (Part D). In all fermentation experiments, the seed culture of the strain DSM 13528 was inoculated by 7.5 mL freezing mid-exponential phase cultures with 30% glycerol, which were stored at −80 °C before use. Then, to obtain the seed culture, a 250 mL screw-cap bottle with a 75 mL working volume of modified DSMZ 879 medium was cultured at 37 °C for 2 days in a rotary shaker (HYG-A, Taicang Experimental Equipment Factory, Taicang, China) at 150 rpm. Batch fermentation was performed in a 250 mL screw-cap bottle with a 50 mL working volume of modified DSMZ 879 medium, 5 mL seed culture, and the addition of 4 g/L CaCO_3_. After sterilization, the medium was aerated with N_2_ for 1 h between 85 and 100 °C at 1.5 L/min. Then, the medium temperature was dropped to 37 °C. After inoculation, the gas in the headspace was substituted by H_2_ as required, with a pressure of 0.8 bar, and the gas in the headspace was exchanged with fresh syngas every two days. Then, for the bioreactor culture, the seed culture broth (150 mL) was transferred to a 2.7 L bioreactor (BioFio^®^110, New Brunswick Scientific, Enfield, CT, USA) with a 1500 mL working volume of modified DSMZ 879 medium. The temperature and stirring speed in the bioreactor were kept at 37 °C and 200 rpm, respectively. Fermentation was carried out under the completely closed exhaust pipe case, and the gas in the headspace of the bioreactor was kept at 0.8 bar with H_2_, which entered the bioreactor through a microflowmeter. Sugarcane molasses was kindly provided by a local sugar mill in Zhanjiang, China. The raw molasses was firstly diluted using deionized water (mass ratio, molasses/water = 1:2) to form a concentrated solution, and the compositions of the diluted molasses were reported in our previous work [27,28] as follows: conductivity 57.3 ms/cm, sucrose 159 g/L, reducing sugars (i.e., the sum of fructose and glucose) 55 g/L, brix 27.6%, K^+^ 13.45 g/L, Cl^−^ 6.02 g/L, SO_4_^2−^ 7.41 g/L, Na^+^ 1.27 g/L, Ca^2+^ 1.06 g/L, and Mg^2+^ 1.42 g/L. Then, the concentrated solution was further diluted using deionized water (*v*/*v*) as necessary. After sterilization, 35 g/L total sugars from the molasses contained sucrose 23.23 g/L, glucose 3.94 g/L, and fructose 7.97 g/L. To evaluate substrate (i.e., sugar in molasses) inhibition on 2,3-BDO production (Figure 6b), simulated sugar concentrations of 0, 20, 40, 60, 80, and 100 g/L in molasses were further added to the molasses containing 35 g/L total sugars.

To recycle cells of *C. ljungdahlii* and remove the produced 2,3-BDO and high concentration of salts, 2,3-BDO production in a membrane-integrated repeated-batch culture was carried out. The culture conditions were the same as those in the batch culture. The main difference was that no cells were discharged. The cells in the discharged broth were recovered using a 300 kDa CéRAM INSIDE tubular ceramic module membrane with an effective surface area of 0.16 m^2^. The cells were then recycled and passed into the bioreactor. A schematic of the membrane system is shown in Supplementary Material Figure S1.

### 3.2. Analytical Methods

First, 5 mL samples were withdrawn from the culture every 6 h or 12 h for cell density monitoring and product analysis in the bioreaction test. For screw-cap bottle tests, the samples were withdrawn after culturing for 7 days. The concentrations of fructose, sucrose, glucose, ethanol, acetic acid, and 2,3-butanediol were measured by a HPLC apparatus (LC-20AT, Shimadzu, Kyoto, Japan) equipped with an Aminex HPX-87H ion exclusion column and refractive index detector (RI). The process was performed at a temperature of 50 °C and a flow rate of 0.6 mL/min, with 5 mmol/L H_2_SO_4_ as the moving phase. The growth of *C. ljungdahlii* was monitored using a UH5300 spectrophotometer (Hitachi high-tech science corporation, Tokyo, Japan) to measure the optical densities at 600 nm. To further evaluate whether the metabolic pathways shifted, genome information for *C. ljungdahlii* was detected. Analysis of genome-wide differential message RNA (mRNA) expression provides us with greater insights into biological pathways and molecular mechanisms that regulate cell fate and development. With a dynamic range to detect subtle changes in expression level in a hypothesis neutral environment, next-generation sequencing enables an understanding of the biological response to stimuli or environmental changes. Cell pellets from cultures in the bioreactor were collected by centrifugation at 10,000× *g* under −4 °C for 10 min at 24 h, frozen in liquid nitrogen immediately, and stored at −80 °C. The mRNA isolation and high-throughput mRNA sequencing (RNA-Seq) were performed by Majorbio (Beijing, China). Total RNA was extracted using the TruSeqTM Stranded Total RNA Library Prep Kit (Ambion, Santa Clara, CA, USA) following the manufacturer’s protocol. RNA integrity was evaluated using the Agilent 2100 Bio-analyzer (Agilent Technologies, Santa Clara, CA, USA). The samples with RNA Integrity Number (RIN) ≥ 7 were subjected to subsequent analysis. The libraries were constructed using the TruSeq Stranded mRNA LTSample Prep Kit (Illumina, San Diego, CA, USA) according to the manufacturer’s instructions. Then, these libraries were sequenced on the Illumina sequencing platform (HiSeqTM 2500) and 150 bp/125 bp paired-end reads were generated. Based on reads per kilobase of transcript per million mapped reads (RPKM) normalization, the gene expression profiles were analyzed. The differential genes were analyzed using Bioconductor edgeR (V3.4.6); information was from Clusters of Orthologous Groups (COG, https://www.ncbi.nlm.nih.gov/research/cog/api/cog/ accessed on 5 January 2022) and Kyoto Encyclopedia of Genes and Genomes (KEGG, http://www.genome.jp/kegg/ accessed on 5 January 2022). Meanwhile, KEGG annotation results were derived from the KAAS (KEGG Automatic Annotation Server). Statistical analysis of the different experimental groups was conducted by subjecting the experimental data to one-way analysis of variance (ANOVA) using OriginPro 2018 software (Origin Lab Corporation, Northampton, MA, USA) at a 95% confidence level. The data presented in the figures are the average values with error bars.

Flux (J) represents the work efficiency, which can express the filtration ability of a membrane.

(1) $$J=\frac{V_{}}{A\cdot t}$$

where V is the total volume of permeate, A is the effective area of the membrane, t is the filtration time.

According to Lim et al. [44], the membrane fouling model was given as follows:

Membrane resistance:

1/J = 1/J_0_ + K_m_t (2)

Pore blocking resistance:

lnJ = −K_p_t + ln J_0_ (3)

Cake resistance:

1/J^2^ = 1/J_0_^2^ + K_c_t (4)

where K_m_, K_p_, and K_c_ are system parameters relating to membrane resistance, pore blocking resistance, and cake resistance, respectively. J_0_ is the initial permeate flux.

## 4. Conclusions

For the first time, sole cane molasses without the addition of other ingredients was used for 2,3-BDO production by nonpathogenic *C. ljungdahlii*. However, the cell growth was significantly inhibited by KCl, and sugars above 35 g/L inhibited cell growth and 2,3-BDO production. More seriously, 2,3-BDO production was inhibited by itself. As a result, an initial value of 35 g/L total sugars in cane molasses was suitable for 2,3-BDO production in batch culture, and the obtained concentrations of 2,3-BDO and ethanol were 11.1 and 4.90 g/L, respectively. Finally, an integrated fermentation and membrane separation process was developed to maintain high 2,3-BDO production.

## Figures and Tables

**Table 1:** The components of M1, M2, M3, M4, M5, and M6.

**Medium ^a^**	**C** **omponents**
M1 ^b^	35 g/L total sugars from molasses; 1.0 g/L NH_4_Cl, 0.1 g/L KCl, 0.2 g/L MgSO_4_·7 H_2_O, 0.8 g/L NaCl, 0.02 g/L CaCl_2_·2 H_2_O, 0.1 g KH_2_PO_4_, 2.5 mg/L Na_2_WO_4_·2 H_2_O, 1.0 g/L NaHCO_3_, 1.0 g/L cysteine-HCl·H_2_O, 1 g/L yeast extract, 0.5 g/L cysteine, 0.5 mg/L resazurin (i.e., Part A in the modified DSMZ 879 medium).
M2	35 g/L total sugars from molasses; 10 mL vitamin solution (i.e., Part B in the modified DSMZ 879 medium). The vitamin solution contains 2 mg biotin, 2 mg folic acid, 10 mg pyridoxine-HCl, 25 mg thiamine-HCl·2 H_2_O, 5 mg riboflavin, 5 mg nicotinic acid, 5 mg d-Ca-pantothenate, 0.1 mg vitamin B_12_, 5 mg ρ-aminobenzoic acid, and 5 mg lipoic acid in 1 L distilled water
M3	35 g/L total sugars from molasses; 10 mL trace element solution (i.e., Part C in the modified DSMZ 879 medium). The trace element solution contains 2.0 g nitrilotriacetic acid, 1.3 g MnCl_2_·H_2_O, 0.4 g FeSO_4_·7 H_2_O, 0.2 g CoCl_2_·7 H_2_O, 0.2 g ZnSO_4_·7 H_2_O, 0.2 g Na_2_MoO_4_·2 H_2_O, 0.02 g NiCl_2_·6 H_2_O, and 0.1 g Na_2_SeO_3_·5 H_2_O in 1 L distilled water.
M4	35 g/L total sugars from molasses; Part A, Part B, Part C in the modified DSMZ 879 medium
M5	35 g/L total sugars from molasses; 1.0 g/L NH_4_Cl, 0.1 g/L KCl, 0.2 g/L MgSO_4_·7 H_2_O, 0.8 g/L NaCl, 0.02 g/L CaCl_2_·2 H_2_O, 0.1 g KH_2_PO_4_, 2.5 mg/L Na_2_WO_4_·2 H_2_O, 1.0 g/L NaHCO_3_, 1 g/L yeast extract, 0.5 mg/L resazurin (Part A), 10 mL trace element solution (Part B), and 10 mL vitamin solution (Part C) in the modified DSMZ 879 medium
M6	The molasses contained a final concentration of 35 g/L total sugars

^a^ The molasses was not pretreated; ^b^ The whole components in the molasses were used, and the final concentration of the total sugars was 35 g/L.

## Data Availability

The data presented in this study are available on request from the corresponding author.

## Supplementary Materials

The following supporting information can be downloaded at online, Figure S1: Schematic diagram of the membrane system for 2,3-BDO production in a membrane bioreactor; The detailed information of the modified DSMZ 879 medium.

## Sample Availability

Samples of the compounds are available from the authors.
