# Supplementary material for: Effectively Converting Cane Molasses into 2,3-Butanediol Using Clostridium ljungdahlii by an Integrated Fermentation and Membrane Separation Process
Source: Molecules. 2022 Jan 30;27(3):954. doi: 10.3390/molecules27030954 (PMC8839846; doi:10.3390/molecules27030954)
Supplement: Supplementary file 1 [file molecules-27-00954-s001.zip › molecules-1570802-supplementary.pdf]

Effectively converting cane molasses into 2,3-butanediol using *Clostridium ljungdahlii* by an integrated fermentation and membrane separation process

Yuling Yang 1,2, Tingting Deng 3, Weifeng Cao 2,4,, Fei Shen 2,4, Sijia Liu 1, Jing Zhang 1, Xinquan Liang 1,* and Yinhua Wan 2,4*

[1] Department of Sugar Engineering, College of Light Industry and Food Engineering, Guangxi University, Nanning 530004, China; yangyulingmie@163.com (Y.Y.); lsj53158352@163.com (S.L.); zhangjing0542@163.com (J.Z.)
[2] State Key Laboratory of Biochemical Engineering, Institute of Process Engineering, Chinese Academy of Sciences, Beijing 100190, China; fshen@ipe.ac.cn (F.S.); yhwan@ipe.ac.cn (Y.W.)
[3] Agro-product Safety Research Center, Chinese Academy of Inspection and Quarantine, Beijing 100123, China; angellove0405@163.com
[4] School of Chemical Engineering, University of Chinese Academy of Sciences, Beijing 100049, China
* Correspondence: wfcao@ipe.ac.cn (W.C.); lxq10713@163.com (X.L.); Tel./Fax: +86-1062650673 (W.C.)

1. The modified DSMZ 879 medium

The modified DSMZ 879 medium with the following composition (per liter): 5.0 g fructose, 1.0 g NH_4Cl, 0.1 g KCl, 0.2 g $MgSO_4 \cdot 7\ H_2O$, 0.8 g NaCl, 0.02 g $CaCl_2 \cdot 2\ H_2O$, 0.1 g KH_2PO_4, 2.5 mg $Na_2WO_4 \cdot 2\ H_2O$, 1.0 g $NaHCO_3$, 1.0 g cysteine-HCl·H_2O, 1 g yeast extract, 0.5 g cysteine, 0.5 mg resazurin, 10 mL trace element solution and 10 mL vitamin solution. Trace element solution contains 2.0 g nitrilotriacetic acid, 1.3 g $MnCl_2 \cdot H_2O$, 0.4 g $FeSO_4 \cdot 7\ H_2O$, 0.2 g $CoCl_2 \cdot 7\ H_2O$, 0.2 g $ZnSO_4 \cdot 7\ H_2O$, 0.2 g $Na_2MoO_4 \cdot 2\ H_2O$, 0.02 g $NiCl_2 \cdot 6\ H_2O$ and 0.1 g $Na_2SeO_3 \cdot 5\ H_2O$ in 1 L distilled water. Vitamin solution involves 2 mg biotin, 2 mg folic acid, 10 mg pyridoxine-HCl, 25 mg thiamine-HCl·2 H_2O, 5 mg riboflavin, 5 mg Nicotinic acid, 5 mg D-Ca-pantothenate, 0.1 mg vitamin B_{12}, 5 mg ϱ-aminobenzoic acid and 5 mg lipoic acid in 1 L distilled water. Analytical grade

chemicals used in the medium were purchased from Sinopharm Chemical Reagent Co., Ltd. (Shanghai, China). The medium was assembled in anaerobic chamber (Ruskinn technology Ltd., Sony Technology center, Pencoed, Bridgend Mid Glamorgan, UK). After autoclaving, $FeSO_4$, vitamins, cysteine-HCl and $NaHCO_3$ were added using syringe with 0.2 μm filter.

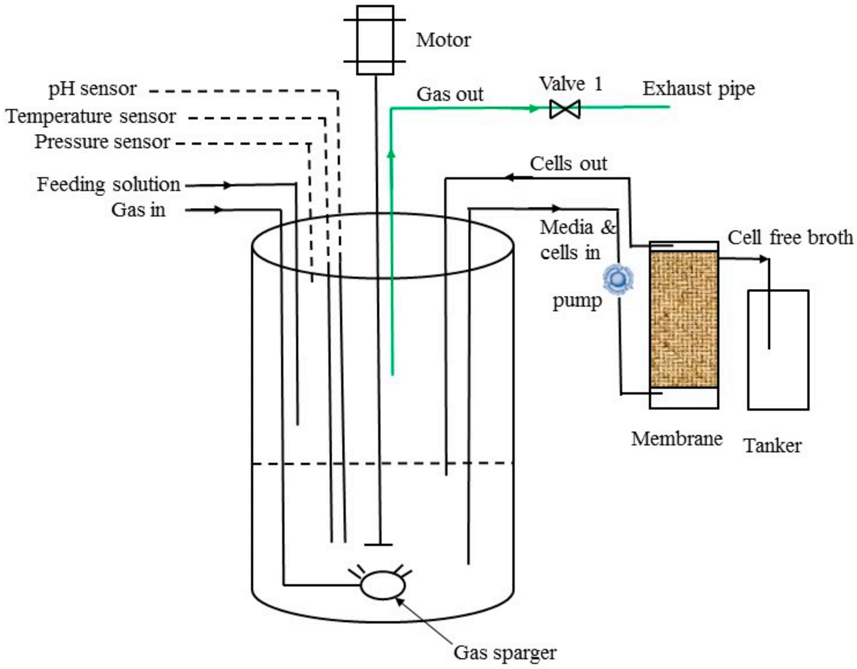

Figure S1. Schematic diagram of the membrane system for 2,3-BDO production in a membrane bioreactor.
